# Supplementary figures and images for: Identifying weather patterns affecting household date palm sap consumption in Bangladesh, 2013–2016
Source: PLoS One. 2024 Nov 20;19(11):e0313904. doi: 10.1371/journal.pone.0313904 (PMC11578510; doi:10.1371/journal.pone.0313904)

## NOAA Weather Stations

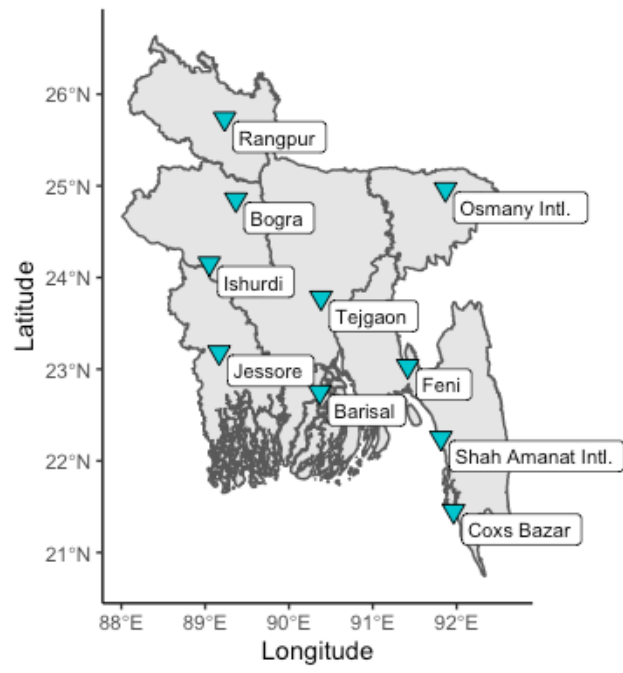

Supplement: S1 Fig — (PDF) [file pone.0313904.s001.pdf]

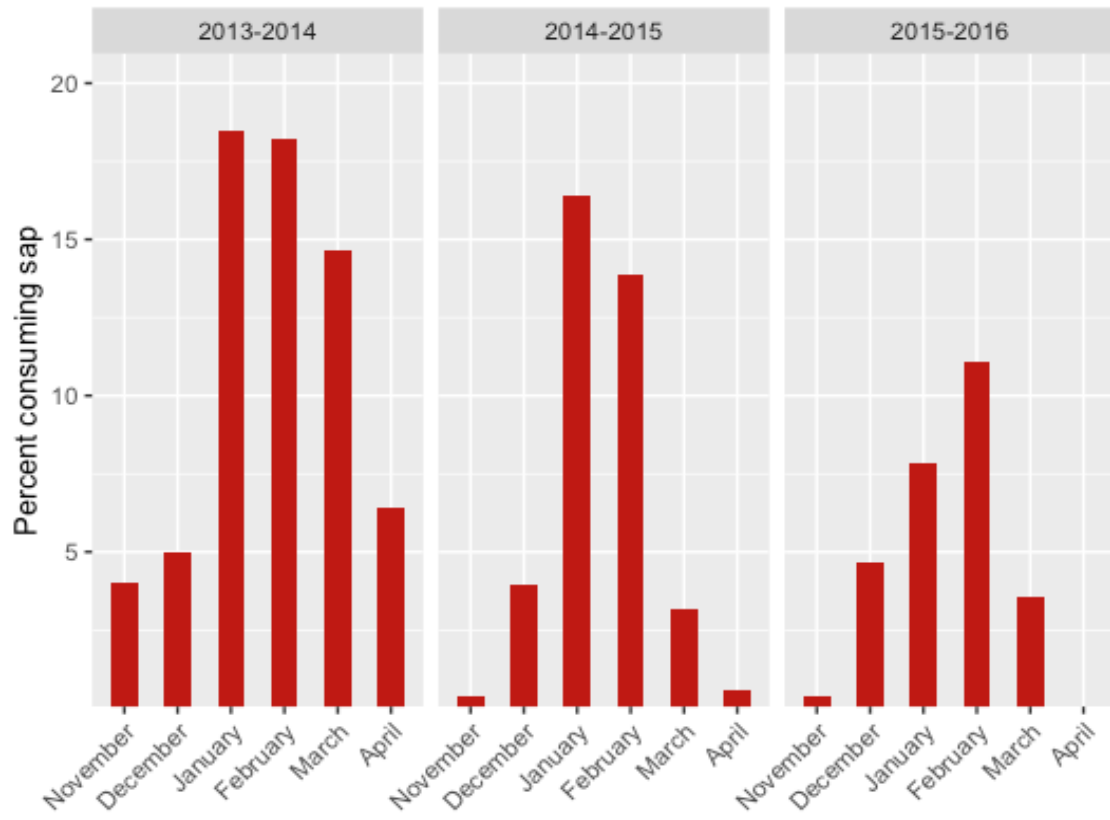

Supplement: S2 Fig — (PDF) [file pone.0313904.s002.pdf]

A

Average visibility Nov. - Apr., 2013 – 2016

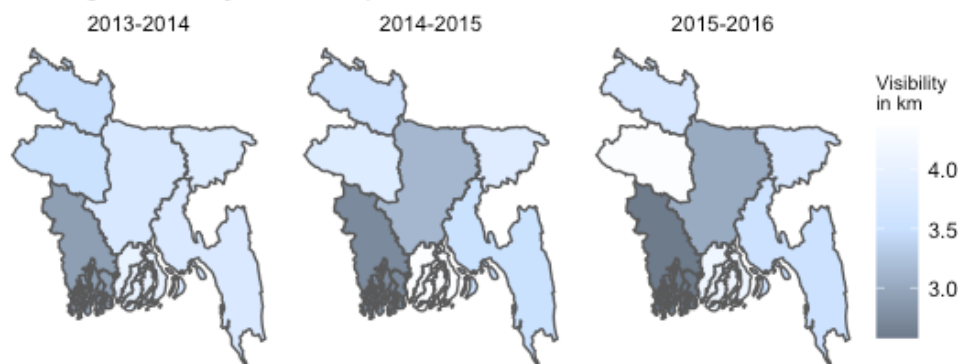

B

Average relative humidity Nov. - Apr., 2013 – 2016

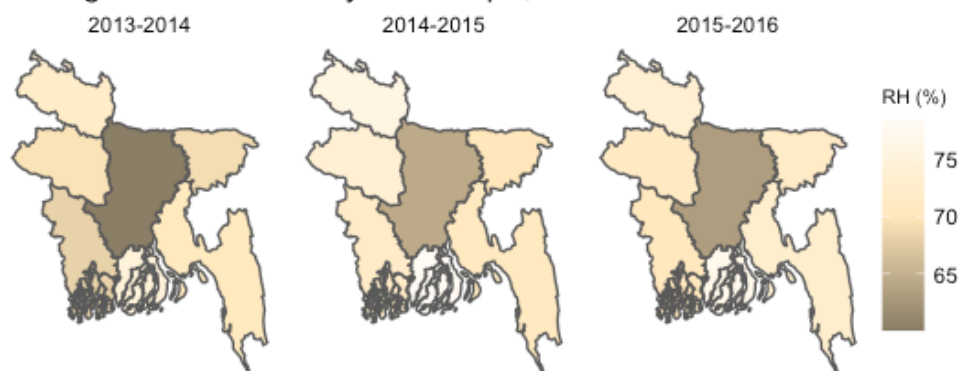

Supplement: S3 Fig — (A) Average of all daily values of visibility in kilometers over sap seasons by division (B) Average of all daily percentages of relative humidity over sap seasons by division. (PDF) [file pone.0313904.s003.pdf]
