# Supplementary material for: Identifying weather patterns affecting household date palm sap consumption in Bangladesh, 2013–2016
Source: PLoS One. 2024 Nov 20;19(11):e0313904. doi: 10.1371/journal.pone.0313904 (PMC11578510; doi:10.1371/journal.pone.0313904)
Supplement: S4 Fig — (A) Receiver operating curve of logistic regression model of month, location (administrative division), and 30-day rolling averages of minimum temperature and cumulative precipitation on date palm sap consumption, (B) Receiver operating curve of logistic regression model of month, location (administrative division), and sap season on date palm sap consumption, (C) Receiver operating curve of logistic regression model of month, location (administrative division), 30-day rolling averages of minimum temperature and cumulative precipitation, and sap season on date palm sap consumption. (PDF) [file pone.0313904.s004.pdf]

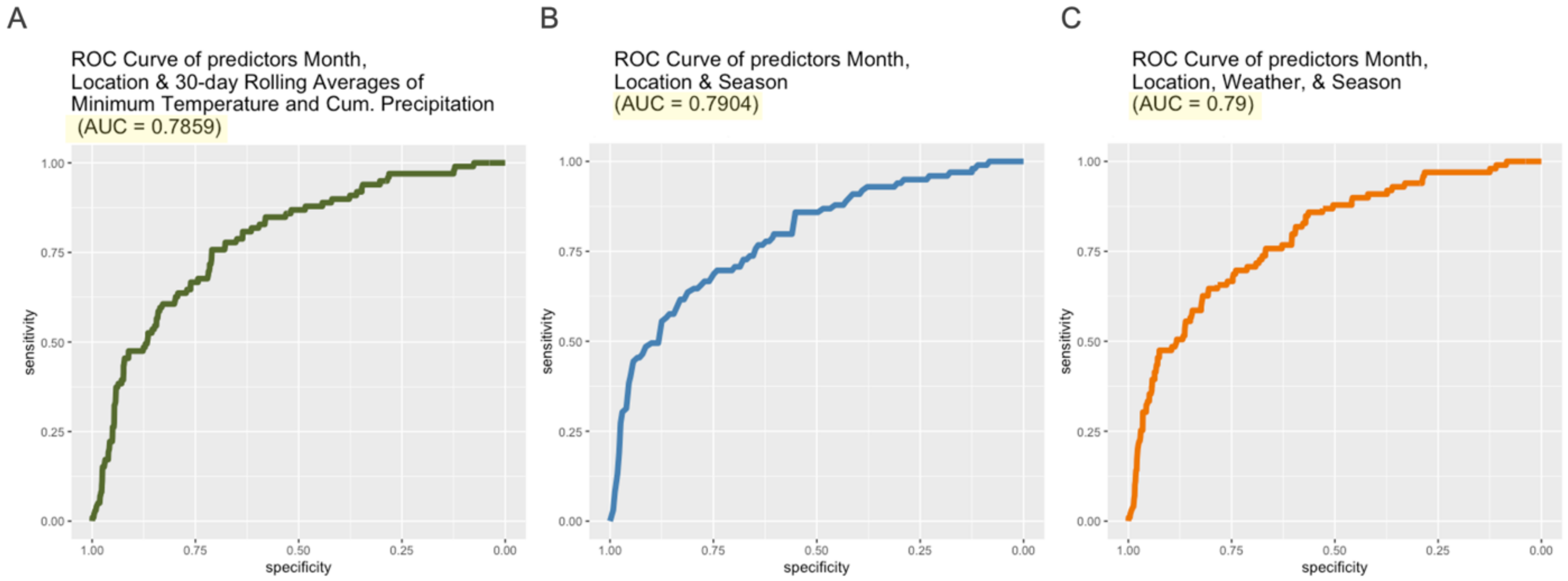
A
ROC Curve of predictors Month, Location & 30-day Rolling Averages of Minimum Temperature and Cum. Precipitation
(AUC = 0.7859)
B
ROC Curve of predictors Month, Location & Season
(AUC = 0.7904)
C
ROC Curve of predictors Month, Location, Weather, & Season
(AUC = 0.79)
sensitivity
specificity
1.00
0.75
0.50
0.25
0.00
