# Supplementary material for: Identifying weather patterns affecting household date palm sap consumption in Bangladesh, 2013–2016
Source: PLoS One. 2024 Nov 20;19(11):e0313904. doi: 10.1371/journal.pone.0313904 (PMC11578510; doi:10.1371/journal.pone.0313904)
Supplement: S1 Table — (PDF) [file pone.0313904.s005.pdf]

**Table S1**: $\chi^2$ Likelihood ratio tests to determine contribution of extended covariates to specified null models for household date palm sap consumption in the past month in responses given November- April 2013-2016 in Bangladesh.

| **Null** | **Extended** | **DF** | **LR $\chi^2$** |
|---|---|---|---|
| month + division | **+ season** | 1 | 56.36 *** |
| month + division + season | **+ cat_mintemp** | 1 | 1.31 |
| month + division + season | **+ SMA_mintemp** | 1 | 7.74 ** |
| month + division + season | **+ cat_precip** | 1 | 14.69 *** |
| month + division + season | **+ SMA_precip** | 1 | 5.15 * |
| month + division + season + SMA_mintemp | **+ cat_precip** | 1 | 13.75 *** |
| month + division + season + SMA_mintemp | **+ SMA_precip** | 1 | 8.00 ** |
| month + division + season + cat_precip | **+ SMA_mintemp** | 1 | 6.80 ** |
| month + division + season + SMA_precip | **+ SMA_mintemp** | 1 | 10.59 ** |
| month + division + season | **+ cat_visibility** | 1 | 0.23 |
| month + division + season | **+ SMA_visibility** | 1 | 2.86 |
| month + division + season | **+ cat_relativehumidity** | 1 | 1.13 |
| month + division + season | **+ SMA_relativehumidity** | 1 | 2.35 |

*= significant at $p < 0.05$
** = significant at $p < 0.01$
*** = significant at $p < 0.001$

cat: categorical monthly average of daily values for the weather variable within the division
SMA: simple moving average of daily values for the weather variable in the division within the 30 days preceding the response
