# Supplementary material for: Identifying weather patterns affecting household date palm sap consumption in Bangladesh, 2013–2016
Source: PLoS One. 2024 Nov 20;19(11):e0313904. doi: 10.1371/journal.pone.0313904 (PMC11578510; doi:10.1371/journal.pone.0313904)
Supplement: S2 Table — (PDF) [file pone.0313904.s006.pdf]

Table S2: Comparison of candidate logistic models of specified variables on date palm sap consumption using AICc

Covariates	K	AICc	Δ AICc	Log-Likelihood
month + division + season + SMA_mintemp + SMA_precip	16	**2119.19**	-	-1043.54
month + division + season	14	**2130.91**	11.71	-1051.41
month + division + SMA_mintemp + SMA_precip	14	**2133.98**	14.79	-1052.95
month + division	12	**2183.25**	64.46	-1079.59

SMA: simple moving average of daily values for the weather variable in the division within the 30 days preceding the response
